# The Association of Broadband Internet Use With Drug Overdose Mortality Rates in the United States: Cross-Sectional Analysis

**DOI:** 10.2196/52686

**Published:** 2024-06-26

**Authors:** Ioannis Karakis, Genti Kostandini, Konstantinos Tsamakis, Velma Zahirovic-Herbert

**Affiliations:** 1 Department of Neurology Emory University School of Medicine Atlanta, GA United States; 2 Department of Neurology University of Crete School of Medicine Heraklion Greece; 3 Department of Agricultural and Applied Economics University of Georgia Athens, GA United States; 4 Institute of Psychiatry, Psychology and Neuroscience King’s College London London United Kingdom; 5 Institute of Medical and Biomedical Education St George’s, University of London London United Kingdom; 6 Second Department of Psychiatry National and Kapodistrian University of Athens Attikon University General Hospital Athens Greece; 7 Department of Finance, Insurance, and Real Estate Fogelman College of Business and Economics University of Memphis Memphis, TN United States

**Keywords:** opioids, broadband internet, mortality, public health, digital divide, access, availability, causal, association, correlation, overdose, drug abuse, addiction, substance abuse, demographic, United States, population

## Abstract

**Background:**

The availability and use of broadband internet play an increasingly important role in health care and public health.

**Objective:**

This study examined the associations between broadband internet availability and use with drug overdose deaths in the United States.

**Methods:**

We linked 2019 county-level drug overdose death data in restricted-access multiple causes of death files from the National Vital Statistics System at the US Centers for Disease Control and Prevention with the 2019 county-level broadband internet rollout data from the Federal Communications Commission and the 2019 county-level broadband usage data available from Microsoft’s Airband Initiative. Cross-sectional analysis was performed with the fixed-effects regression method to assess the association of broadband internet availability and usage with opioid overdose deaths. Our model also controlled for county-level socioeconomic characteristics and county-level health policy variables.

**Results:**

Overall, a 1% increase in broadband internet use was linked with a 1.2% increase in overall drug overdose deaths. No significant association was observed for broadband internet availability. Although similar positive associations were found for both male and female populations, the association varied across different age subgroups. The positive association on overall drug overdose deaths was the greatest among Hispanic and Non-Hispanic White populations.

**Conclusions:**

Broadband internet use was positively associated with increased drug overdose deaths among the overall US population and some subpopulations, even after controlling for broadband availability, sociodemographic characteristics, unemployment, and median household income.

## Introduction

Over 900,000 individuals have died from drug overdoses within the last 2 decades, posing a significant public health concern in the United States [1,2]. Opioids, either obtained illicitly or as a prescription, accounted for approximately 75% of the total drug overdose deaths in 2020 [1,2]. Recent numbers suggest an acceleration of both drug and opioid overdose deaths during the COVID-19 pandemic, with the United States experiencing the largest number of drug overdose deaths during this period (93,300 deaths) compared to that reported in any given year within the last 2 decades.

Internet access alongside digital literacy in general have been increasingly recognized as a “super” social determinant of health [3,4]. Access to broadband internet has been identified as a crucial public health issue (including its effect on the domain of access to credible information [5]) and a critical infrastructure for equitable access to health care, especially for underserved communities [6,7]. More specifically, the widening availability of broadband internet could potentially worsen the health inequalities in the United States, as it disproportionately impacts already marginalized groups such as racial/ethnic minorities, the older population, individuals with lower incomes, those with less education, and residents of rural areas [5]. According to the Federal Communications Commission (FCC), approximately 19 million individuals in the United States do not have access to a reliable broadband service, often referred to as reliable high-speed internet. This issue, termed the digital divide, primarily targets rural regions but also encompasses “segments of segregated urban areas that remain disconnected” [4]. Individuals with reduced income and less formal education are less inclined to possess a home broadband service or a mobile data plan subscription, necessitating their reliance on limited cell plan data or local public Wi-Fi hot spots [8,9].

Broadband internet access has been linked with disparities in various health outcomes, ranging from health information–seeking and health communication [10] to access to care during public health emergencies requiring remote care [11]. A recent US study demonstrated an association between lack of broadband coverage and adverse mental health outcomes [12]. In addition, access to broadband internet could affect drug and substance use through several channels. For instance, purchase of drugs via the internet is faster, anonymous, and less risky, which makes overall access to drugs easier; accordingly, the internet has been deemed a “pipeline for narcotics” [13]. Existing research indicates that in instances where drugs are easily accessible on the internet, there is a notable surge in the number of new and first-time users regardless of socioeconomic status [13]. Although, theoretically, increases in knowledge and information should lead to more optimal consumer choices, substantial networking opportunities may result in peers having a significant influence over health behaviors [14]. This raises the possibility that the marked growth in US drug abuse may have partially stemmed from the wider availability of illicit drugs on the internet [15].

The availability of telehealth through broadband internet is likely to reduce health disparities by connecting providers with individuals living in remote areas [6,7,16]. Telehealth brings specialized health care to communities where it was previously unavailable [17,18]. Thus, having access to and the ability to use broadband internet services can affect major public health outcomes such as drug overdose deaths.

Furthermore, access and usage data sets are critically important in building a full and accurate broadband internet map. Access data show current and future plans, while usage data help to understand how access translates into consumption. The aim of our study was to explore how broadband access and usage intersect with drug abuse by using access and usage data to elucidate the association of broadband internet with drug overdose mortality rates. In addition, given that in this time and age almost all counties have internet/mobile data plans in place, our study aimed to explore whether the emphasis should shift from broadband availability to actual usage, which is potentially the more relevant metric from a public health perspective.

## Methods

### Data Sources

Primary data for the study were obtained from three main sources: (1) 2019 county-level drug overdose death data from restricted-access multiple causes of death files available from the National Vital Statistics System (NVSS) at the US Centers for Disease Control and Prevention (CDC); (2) 2019 county-level broadband internet rollout data from the FCC; and (3) 2019 county-level broadband usage data available from Microsoft’s Airband Initiative. In addition, county-level socioeconomic and demographic characteristics were gathered from the US Census Bureau and US Bureau of Labor Statistics.

All drug overdose deaths were measured per 10,000 persons per year by county. Following the CDC and NVSS guidelines, we used the Tenth Revision of the *International Classification of Diseases Clinical Modification* codes X40-X44 (unintentional), X60-X64 (intentional), X85 (homicide), and Y10-Y14 (undetermined) to identify all drug overdose deaths.

### Ethical Considerations

As this was a secondary data analysis, there was no requirement of approval from an institutional review board.

### Statistical Analysis

Data analysis was performed using descriptive analysis and a cross-sectional regression analysis. In addition to examining the effects of broadband availability and usage on drug overdose deaths, we investigated how these effects vary based on different subpopulations and location characteristics.

The one-way fixed-effects regression equation used for this analysis was:

*Y_j_* = *α*_0_ + ∂*BB_j_* + *βX_j_* + δ*_s_* + *ε_s_*

where *Y_j_* is the outcome variable for county *j*. The *BB_j_* vector indicates the broadband internet availability and usage in a given county during 2019. Internet availability was measured as the percentage of people per county with access to fixed terrestrial broadband at minimum speeds of 25 Mbps/3 Mbps. Internet usage was measured as the percentage of people per county that use the internet at broadband speeds based on the methodology provided by Microsoft.

Microsoft estimates broadband usage by combining data from multiple Microsoft services. These data are combined with the number of households per county and zip code. While Microsoft suppresses any location with less than 20 devices in zip code–level data, this is not an issue for the county-level data used in our study. Every time a device receives an update or connects to a Microsoft service, Microsoft estimates the throughput speed of a machine. To calculate the broadband speed, they use the size of the package sent to the computer and the total time of the download. They determine the zip code–level location data via the reverse IP. Therefore, they can count the number of devices that have connected to the internet at broadband speed for each zip code based on the FCC’s definition of broadband, which is 25 Mbps per download. The zip code–level data were then aggregated to the county level. Microsoft’s data might be more representative in regions where its products are more widely used and less representative in regions where its products have lower penetration rates. Since Microsoft’s data primarily come from devices running Windows operating systems and services such as Bing, Edge browser, and Xbox, this might skew the data toward certain types of devices and services and may not capture the full spectrum of internet-connected devices or platforms.

The coefficient on these variables (∂) captures the direct effect of broadband availability and usage on the outcome. *X_j_* is a vector of observed socioeconomic and demographic characteristics of county *j* and *ε_s_* is the error term. State fixed effects eliminate the omitted variable bias that may result from the time-invariant differences between states. The analysis was performed using robust Huber-White standard errors to capture arbitrary within-county heteroscedasticity.

## Results

The descriptive statistics showed that 1.71 drug overdose deaths occurred per 10,000 county population in 2019 in the United States. Although approximately 76.6% of people in a given county had access to broadband internet in 2019, only approximately 28% of people were using the internet at broadband speeds (Table 1).

**Table 1:** Summary statistics.

Variable			Counties, n		Mean (SD)
**Outcome variables**					
	Drug overdose deaths^a^	3104		1.71 (1.48)	
	Internet availability (% of county population with access to broadband internet)	3104		76.627 (23.88)	
	Internet usage (% of county population using internet at broadband speeds)	3104		28.133 (19.027)	
	Kidney deaths^a^	3103		2.179 (1.721)	
	Non-Hispanic White deaths^a^	3104		1.917 (1.739)	
	Non-Hispanic Black deaths^a^	3093		2.718 (45.691)	
	Non-Hispanic other deaths^a^	3104		8.354 (35.53)	
	Hispanic deaths^a^	3104		0.844 (3.17)	
	Deaths for those with less than high school education^a^	3104		2.889 (4.101)	
	Deaths for those with some college education^a^	3104		4.173 (4.033)	
	Deaths for those with college education or higher^a^	3103		0.486 (1.446)	
	Male deaths^a^	3104		2.165 (2.138)	
	Female deaths^a^	3104		1.264 (1.446)	
	Deaths among those aged ≤19 years^a^	3103		0.1 (0.447)	
	Deaths among those aged 20-29 years^a^	3103		2.205 (3.554)	
	Deaths among those aged 30-39 years^a^	3104		3.796 (5.117)	
	Deaths among those age 40-49 years^a^	3104		3.244 (4.866)	
	Deaths among those aged 50-64 years^a^	3104		2.39 (3.048)	
	Deaths among those aged ≥65 years^a^	3104		3.108 (12.245)	
**Control variables**					
	Percent county poverty	3103		14.429 (5.772)	
	Percent population with no health insurance	3103		11.903 (5.108)	
	Net migration (the number of immigrants minus the number of emigrants in the county)	3104		192.543 (2937.949)	
	Percent of county population with a college degree or higher	3104		22.018 (9.566)	
	Median household income (US $)	3103		55,707.34 (14,456.62)	
	Percent of county population that is unemployed	3103		3.977 (1.41)	
	Share of population aged 20-34 years	3104		0.18 (0.037)	
	Share of population aged 35-49 years	3104		0.175 (0.019)	
	Share of population aged 50-64 years	3104		0.203 (0.024)	
	Share of population aged ≥65 years	3104		0.198 (0.047)	
	Share of the female population	3104		0.499 (0.022)	
	Share of the Black population	3104		0.094 (0.144)	
	Share of the Hispanic population	3104		0.097 (0.138)	
	Share of the Asian population	3104		0.015 (0.028)	
	Counties with population of ≥50,000 (dummy variable)	3104		0.319 (0.466)	
	Metro counties (dummy variable)	3104		0.374 (0.484)	
	Adjacent to an urban area (dummy variable)	3104		0.424 (0.494)	
	Rural county (dummy variable)	3104		0.201 (0.401)	

^a^Measured per 10,000 of the total or specific subpopulation indicated.

The regression analysis results in Table 2 show that a 1% increase in broadband internet usage is significantly associated with a 1.2% increase in overall drug overdose deaths among the general population.

**Table 2:** Associations between broadband internet usage and drug overdose deaths.^a^

Variables	Drug overdose (full sample)	Drug overdose in counties with a population over 50,000	Drug overdose in counties with a population less than 50,000	Kidney disease–related deaths (full sample)
Internet usage, coefficient (robust SE)	0.012 (0.002)^b^	0.008 (0.004)^c^	0.008 (0.003)^d^	0.004 (0.003)^e^
Internet availability	Yes^f^	Yes	Yes	Yes
Socioeconomic variables	Yes	Yes	Yes	Yes
Demographic variables	Yes	Yes	Yes	Yes
State controls	Yes	Yes	Yes	Yes
Robust standard errors	Yes	Yes	Yes	Yes
Number of observations	3103	990	2113	3103
Adjusted *R*^2^	0.296	0.456	0.232	0.214

^a^The regression analysis includes the set of full control variables shown in Table 1.

^b^*P*<.001.

^c^*P*=.05.

^d^*P*=.007.

^e^*P*=.18.

^f^Indicates inclusion of the variable as a control in the regression model; coefficients for subpopulations and the full set of controls are provided in Multimedia Appendix 1.

Splitting the sample into counties with a population more and less than 50,000 showed that the association result was primarily driven by the relationship between these variables in the less populated counties.

This association could result from the fact that the internet encourages greater consumption of drugs by making illegal drugs more accessible. Internet purchasing is more convenient and less risky because of anonymity. Furthermore, online drug sales could also be less risky for suppliers than offline sales. Thus, it is reasonable to expect broadband internet usage to improve market liquidity and spur both the demand and supply of illicit drugs. If this is the case, we would expect no significant association between broadband internet and mortality rates from chronic conditions. To test our assumption, we considered mortality rates associated with kidney disease, which is a chronic condition and should not be associated with internet availability. There was no association between internet availability/usage rates and kidney disease–related mortality rates (Table 2).

**Figure 1:**
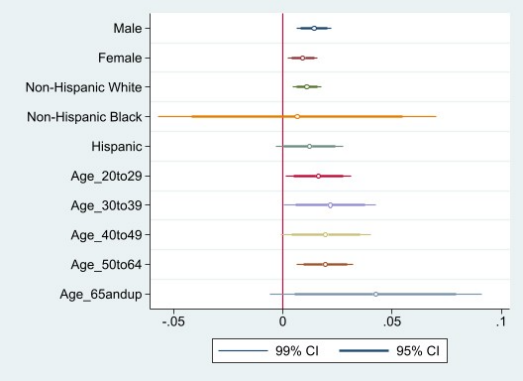
Associations between broadband internet usage and drug overdose deaths for different subpopulations. The figure presents the estimates of regression analysis where each variable is the dependent variable and the estimate shown is the coefficient of internet use as a dummy variable. Each regression includes the full controls shown in Table 1.

Further analysis revealed that different subpopulations are disproportionately affected by the associations between internet usage and drug-related deaths (see Figure 1). More specifically, Figure 1 shows that a 1% increase in internet usage is associated with a 1.1% increase in drug overdose deaths among the non-Hispanic White population, a 1.2% increase among the Hispanic population, a 1.4% increase among the male population, and a 1% increase among the female population. There appears to be no statistically significant association for the Non-Hispanic Black population, although the coefficient’s SE is wide for this population because of the small sample size. Additionally, a 1% increase in broadband internet usage was significantly associated with a 1.7% increase in drug overdose deaths among those aged 20-29 years, a 2.2% increase among those aged 30-39 years, a 2% increase among those aged 40-49 years, a 1.9% increase among those aged 50-64 years, and a 4.3% increase among those 65 years and older compared with those 19 years and younger.

## Discussion

### Principal Findings

The findings of this study show that increased usage of broadband internet is positively and significantly associated with increased drug overdose deaths in the general US population as well as in several subpopulation categories, with the largest increase observed among the population 65 years and older compared with those 19 years and younger.

Our approach is novel in carefully distinguishing two different existing definitions of “broadband internet access” that are critical for studying its equity and potential impact on individual and community health outcomes: (1) its physical availability to individuals and communities (broadband availability) and (2) its adoption and effective use by those individuals and communities (broadband usage) [7,19,20]. Although no previous studies have examined the link between broadband internet usage and drug overdose deaths to our knowledge, there have been a few studies examining the link between drug abuse and the internet using the rollout of Craigslist [15], broadband internet subscription rates and opioid prescribing via telemedicine during COVID-19 [21], broadband internet and youth mental health [22], and broadband internet access and treatment admissions to substance use programs [23].

Specifically, one of the earliest studies examining the link between broadband internet access and drug abuse found that every 10% increase in the number of residential high-speed internet lines per capita at the state level was associated with a 1% increase in admissions to treatment programs for substance abuse [23]. Another study, using the phased rollout of Craigslist, a major web-based platform, found that Craigslist’s entry was associated with a 14.9% increase in drug abuse treatment admissions, a 5.7% increase in drug abuse violations, and a 6.0% increase in drug overdose deaths in the United States [15]. Although different in magnitude, these previous findings are in line with the results of this study, especially in terms of the direction of the effects of the internet on the health outcomes examined. Other researchers examining the link between broadband internet and youth mental health found similar directional effects of internet access. In particular, access to high-speed internet resulted in an increase in diagnoses of depression, anxiety, drug abuse, and personality disorders for the younger cohorts of both males and females but not for the older cohort [22]. In contrast, Oyler et al [21] found that broadband subscription rates (low and high) did not affect opioid prescriptions dispensed in Kentucky counties during the time period of Executive Order 2020-243 issued by the state’s governor on March 22, 2020, which limited nonurgent medical procedures in Kentucky to conserve personal protective equipment and medical supplies for patients with COVID-19. These findings suggest that access to broadband internet may not affect legal prescriptions of opioids. Despite the heterogeneity, if taken together with previous research, our findings suggest that broadband internet usage may be contributing to the drug epidemic in the United States.

### Strengths and Limitations

We should acknowledge that our measure of broadband internet availability does not include internet access via mobile phones. Unfortunately, these data are currently not available; thus, our estimation of the effect of broadband availability would be an underestimation. To obtain information on internet access via mobile use, we downloaded the American Community Survey (ACS) census data for 2021 and 2022, which were the two most recent years (along with 2016) with data available on two relevant questions. The first question was whether or not someone in the household uses or connects to the internet, regardless of whether or not they pay for the service. The second was whether or not anyone in the household has a data plan for a smartphone or other mobile device. Since the ACS does not provide the county Federal Information Processing Standard (FIPS) code for all observations, we could only identify the county FIPS codes for a subset of the respondents and only data for 2022 included FIPS codes for rural counties. Using the Economic Research Service–US Department of Agriculture rural-urban continuum codes [24] to classify rural counties, we found that 94.30% (n=21,733) of households in rural counties and 95.37% (n=1,897,961) in metro and urban counties had a household member with a data plan. Using the same sample and definition of rural, we also found that 97.22% (n=22,657) of households in rural counties and 96.71% (n=1,999,196) of households in metro and urban counties had a household member that uses or connects to the internet. Thus, the use of data plans and internet connection in rural areas is very similar to those in metro and urban areas. Another limitation is that we do not have data for a longitudinal study that could provide opportunities for examining causal relationships.

These limitations notwithstanding, the findings of this study underscore the importance of curbing illegal drug sales via web-based pharmacies and social networks. In addition, it is important for the Food and Drug Administration to monitor the shipping of medications to the United States from other countries. The results of this study emphasize the importance of tracking prescription drug use and sales on web-based platforms to better understand the amount and the types of transactions of prescription drugs taking place on these platforms and the role these platforms play in prescription drug abuse in the United States.

Finally, we have included controls in the regression model that mediate the association between internet connectivity and overdose rates, such as the percentage of uninsured population in a county, which, as expected, was negatively associated with overdose rates although not significantly. Even though determining the mediating effects of such variables was not within the scope of this study, research on the mediating effects of insurance availability, distance from medical resources, and other factors may be an important area for future research.

### Conclusions

Broadband internet usage is positively associated with increased drug overdose deaths among the overall US population and some subpopulations, even after controlling for broadband availability, sociodemographic characteristics, unemployment, and median household income. These findings merit further investigation and can assist in policy shaping and thoughtful resource allocation to susceptible populations, especially in areas with recently improved broadband internet access.

## Appendix

Multimedia Appendix 1 Table S1: Associations between broadband internet usage and drug and overdose deaths; Table S2: Associations between broadband internet usage and drug and overdose deaths with coefficients for the full set of controls; Table S3: Associations between broadband internet usage and drug overdose deaths for different subpopulations with coefficients for the full set of controls.

## Abbreviations

ACS: American Community Survey
CDC: Centers for Disease Control and Prevention
FCC: Federal Communications Commission
FIPS: Federal Information Processing Standard
NVSS: National Vital Statistics System
