# Supplementary material for: The Association of Broadband Internet Use With Drug Overdose Mortality Rates in the United States: Cross-Sectional Analysis
Source: Online J Public Health Inform. 2024 Jun 26;16:e52686. doi: 10.2196/52686 (PMC11237777; doi:10.2196/52686)
Supplement: Multimedia Appendix 1 [file ojphi_v16i1e52686_app1.docx]

**Multimedia Appendix 1**

**Table S1: Associations between broadband internet usage and drug and overdose deaths**

|  | Drug overdose  in metro countis | Drug  overdose  in urban counties | Drug overdose  in rural counties |
| --- | --- | --- | --- |
| Internet usage | 0.015^c^ | 0.005 | 0.007 |
|  | (0.0030) | (0.0040) | (0.0070) |
| Internet availability | 0.008^c^ | 0.002 | -0.0007 |
|  | (0.0030) | (0.0020) | (0.0030) |
| Percent county poverty | 0.036^b^ | 0.025^a^ | 0.048^a^ |
|  | (0.0160) | (0.0150) | (0.0260) |
| Percent population with no health insurance | -0.002 | -0.017 | -0.023 |
|  | (0.0220) | (0.0160) | (0.0300) |
| Net migration | 1.48E-07 | 0.0003^b^ | 0.002^b^ |
|  | (0.0000) | (0.0001) | (0.0007) |
| Percent with a college degree or higher | -.018^c^ | -0.007 | 0.004 |
|  | (0.0070) | (0.0090) | (0.0150) |
| Median household income (US dollars) | -1.00e-05^b^ | -.00002^b^ | -0.00002 |
|  | (0.0000) | (0.0000) | (0.0000) |
| Percent unemployed | 0.01 | 0.03 | 0.045 |
|  | (0.0450) | (0.0380) | (0.0680) |
| Share of population in the 20 to 34 years old age group | 7.382^c^ | 1.95 | -2.358 |
|  | (2.4260) | (2.7690) | (6.2680) |
| Share of population in the 35 to 49 years old age group | 12.609^c^ | 17.898^c^ | 6.946 |
|  | (3.4890) | (3.7750) | (5.9740) |
| Share of population in the 50 to 64 years old age group | 13.004^c^ | 7.955^b^ | 10.585^b^ |
|  | (2.9330) | (3.5690) | (4.7510) |
| Share of population that is 65 years old and older | 3.163^a^ | -0.067 | -7.595^b^ |
|  |  | (1.9210) | (2.1570) |
| Share of females | 8.901^c^ | 7.019^c^ | 5.912 |
|  | (2.64) | (2.43) | (4.64) |
| Share of black population | 0.266 | -1.522^c^ | -2.740^c^ |
|  | (0.3650) | (0.4110) | (0.9260) |
| Share of hispanic population | -0.834 | -0.149 | 0.867 |
|  | (0.5190) | (0.3770) | (1.0870) |
| Share of asian population | -1.791 | 5.107 | 5.414 |
|  | (1.4290) | (3.7170) | (14.3530) |
| N | 1161 | 1317 | 625 |
| Adjusted *R*^2^ | 0.433 | 0.272 | 0.161 |

*Note.* Robust standard errors are shown in parentheses.^a^ *p* <0.1; ^b^ *p* < 0.05; ^c^ *p* < 0.01.

Metro, Urban and Rural counties are defined according to the Unites States Department of Agriculture Economic Research Service 2013 Rural-Urban continuum code.

Matro counties are classified as:

1 Counties in metro areas of 1 million population or more

2 Counties in metro areas of 250,000 to 1 million population

3 Counties in metro areas of fewer than 250,000 population

Urban counties are classified as:

4 Urban population of 20,000 or more, adjacent to a metro area

5 Urban population of 20,000 or more, not adjacent to a metro area

6 Urban population of 2,500 to 19,999, adjacent to a metro area

7 Urban population of 2,500 to 19,999, not adjacent to a metro area

Rural counties are classified as:

8 Completely rural or less than 2,500 urban population, adjacent to a metro area

9 Completely rural or less than 2,500 urban population, not adjacent to a metro area

**Table S2. Associations between broadband internet usage and drug and overdose deaths with coefficients for the full set of controls**

|  | Drug overdose  full sample | Drug overdose  in counties with over 50K  population | Drug overdose  in counties with less than 50K  population | Kidney related deaths  full sample |
| --- | --- | --- | --- | --- |
| Internet usage | 0.012^c^ | 0.008^b^ | 0.008^c^ | 0.004 |
|  | (0.0020) | (0.0040) | (0.0030) | (0.0030) |
| Internet availability | 0.002^a^ | 0.014^c^ | 0.0004 | 0.003^b^ |
|  | (0.0010) | (0.0040) | (0.0010) | (0.0020) |
| Percent county poverty | 0.023^b^ | 0.036^b^ | 0.033^c^ | 0.068^c^ |
|  | (0.0090) | (0.0170) | (0.0120) | (0.0110) |
| Percent population with no health insurance | -0.0008 | (0.01) | (0.01) | -.052^c^ |
|  | (0.0120) | (0.0220) | (0.0140) | (0.0140) |
| Net migration | -1.42E-06 | 0.00 | 0.0004^c^ | 6.08E-07 |
|  | (0.0000) | (0.0000) | (0.0001) | (0.0000) |
| Percent with a college degree or higher | -0.004 | -.026^c^ | (0.01) | -.040^c^ |
|  | (0.0050) | (0.0070) | (0.0070) | (0.0060) |
| Median household income (US dollars) | -.00002^c^ | (0.00) | -.00002^c^ | -2.91E-06 |
|  | (0.0000) | (0.0000) | (0.0000) | (0.0000) |
| Percent unemployed | -0.01 | 0.02 | 0.02 | -.071^b^ |
|  | (0.0260) | (0.0440) | (0.0330) | (0.0320) |
| Share of population in the 20 to 34 years old age group | 4.334^b^ | 12.297^c^ | (0.43) | -2.536 |
|  | (1.6980) | (2.7020) | (2.2620) | (2.0870) |
| Share of population in the 35 to 49 years old age group | 12.028^c^ | 20.399^c^ | 8.915^c^ | 2.041 |
|  | (2.2970) | (3.7240) | (2.9160) | (2.8220) |
| Share of population in the 50 to 64 years old age group | 11.961^c^ | 15.683^c^ | 10.982^c^ | 2.088 |
|  | (2.0000) | (3.4300) | (2.4590) | (2.4580) |
| Share of population that is 65 years old and older | -1.796 | 8.697^c^ | -5.617^c^ | 3.704^b^ |
|  | (1.2960) | (2.0670) | (1.6500) | -1.592 |
| Share of females | 8.315^c^ | 8.869^b^ | 5.664^c^ | 4.102^b^ |
|  | (1.59) | (3.47) | (1.97) | (1.9540) |
| Share of black population | -.802^c^ | 0.943^b^ | -1.588^c^ | 0.988^c^ |
|  | (0.2680) | (0.3950) | (0.3620) | (0.3300) |
| Share of hispanic population | -0.298 | (0.45) | (0.28) | -0.23 |
|  | (0.2940) | (0.4970) | (0.3810) | (0.0362) |
| Share of asian population | -2.536^a^ | -2.532^a^ | 6.400^a^ | 0.526 |
|  | (1.3330) | (1.3350) | (3.8100) | (1.6380) |
| N | 3103 | 990 | 2113 | 3103 |
| Adjusted *R*^2^ | 0.296 | 0.456 | 0.232 | 0.214 |

*Note.* Robust standard errors are shown in parentheses.^a^*p* <0.1; ^b^*p* < 0.05; ^c^*p* < 0.01.

**Table S3. Associations between broadband internet usage and drug overdose deaths for different subpopulations with coefficients for the full set of controls.**

|  | Non-hispanic (NH) white deaths (per 10K NH white pop) | Non-hispanic (NH) black deaths (per 10K NH black pop) | Hispanic deaths (per 10K Hispanic pop) | Non-hispanic (NH) other deaths (per 10K NH other pop) | Male deaths (per 10K male population) | Female deaths (per 10K female population) |
| --- | --- | --- | --- | --- | --- | --- |
| Internet usage | 0.011^c^ | 0.007 | 0.012^b^ | 0.069 | 0.014^c^ | 0.01^c^ |
|  | (0.0030) | (0.0820) | (0.0060) | (0.0560) | (0.0030) | (0.0020) |
| Internet availability | 0.004^b^ | -.082^a^ | 0.0005 | -.142^c^ | 0.003^a^ | 0.001 |
|  | (0.0020) | (0.0470) | (0.0030) | (0.0320) | (0.0020) | (0.0010) |
| Percent county poverty | -0.014 | 0.158 | -0.018 | 0.168 | 0.03^b^ | 0.014 |
|  | (0.0110) | (0.3380) | (0.0230) | (0.2340) | (0.0130) | (0.0100) |
| Percent population with no health insurance | -0.003 | -0.029 | -0.006 | 0.471 | 0.013 | -0.016 |
|  | (0.0140) | (0.4240) | (0.0290) | (0.2930) | (0.0170) | (0.0130) |
| Net migration | 1.72E-06 | 0.0002 | -3.60E-06 | 0.00009 | -3.33E-07 | -3.27E-06 |
|  | (0.0000) | (0.0003) | (0.0000) | (0.0002) | (0.0000) | (0.0000) |
| Percent with a college degree or higher | -.011^a^ | 0.776^c^ | -.025^a^ | 0.327^b^ | 0.003 | -.012^b^ |
|  | (0.0060) | (0.1890) | (0.0130) | (0.1300) | (0.0070) | (0.0060) |
| Median household income (US dollars) | -.00002^c^ | -0.0002 | -6.56E-06 | -0.00004 | -.00002^c^ | -.00002^c^ |
|  | (0.0000) | (0.0002) | (0.0000) | (0.0001) | (0.0000) | (0.0000) |
| Percent unemployed | 0.012 | -0.818 | -.185^c^ | -1.301^b^ | -0.008 | -0.013 |
|  | (0.0310) | (0.9530) | (0.0650) | (0.6590) | (0.0380) | (0.0280) |
| Share of population in the 20 to 34 years old age group | 6.068^c^ | -104.954^a^ | 8.948^b^ | -96.405^b^ | 4.689^a^ | 4.042^b^ |
|  | (2.0230) | (62.3480) | (4.2620) | (43.1020) | (2.4790) | (1.8600) |
| Share of population in the 35 to 49 years old age group | 12.591^c^ | -228.454^c^ | 1.673 | -310.689^c^ | 14.412^c^ | 9.540^c^ |
|  | (2.7350) | (84.8830) | (5.7630) | (58.2850) | (3.3520) | (2.5150) |
| Share of population in the 50 to 64 years old age group | 14.206^c^ | 82.182 | 7.548 | 112.737^b^ | 16.384^c^ | 7.319^c^ |
|  | (2.3820) | (74.1760) | (5.0200) | (50.7630) | (2.9190) | (2.1900) |
| Share of population that is 65 years old and older | -1.218 | -128.497^c^ | 1.873 | -8.767 | -3.677^a^ | 0.11 |
|  | (1.5430) | (48.0780) | (3.2520) | (32.8890) | (1.8910) | (1.4190) |
| Share of females | 7.956^c^ | -161.417^c^ | 9.107^b^ | -177.257^c^ | 12.811^c^ | 4.469^b^ |
|  | (1.89) | (58.54) | (3.99) | (40.36) | (2.32) | (1.74) |
| Share of black population | 1.455^c^ | -4.236 | 1.755^c^ | 14.919^b^ | -0.635 | -.939^c^ |
|  | (0.3200) | (9.8300) | (0.6740) | (6.8120) | (0.3920) | (0.2940) |
| Share of hispanic population | 0.775^b^ | -6.024 | 1.088 | -5.582 | -0.388 | -0.167 |
|  | (0.3500) | (10.8050) | (0.7380) | (7.4680) | (0.4290) | (0.3220) |
| Share of asian population | -0.658 | -120.727^b^ | -0.92 | 39.18 | -3.782^a^ | -1.207 |
|  | (1.5880) | (48.8630) | (3.3460) | (33.8330) | (1.9460) | (1.4600) |
| N | 3103 | 3093 | 3103 | 3103 | 3103 | 3103 |
| Adjusted *R*^2^ | 0.277 | 0.01 | 0.035 | 0.21 | 0.282 | 0.117 |

*Note.* Robust standard errors are shown in parentheses.^a^*p* <0.1; ^b^*p* < 0.05; ^c^*p* < 0.01.
